# Differences and Influencing Factors of Relative Poverty of Urban and Rural Residents in China Based on the Survey of 31 Provinces and Cities

**DOI:** 10.3390/ijerph19159015

**Published:** 2022-07-25

**Authors:** Hong Sun, Xiaohong Li, Wenjing Li, Jun Feng

**Affiliations:** 1 School of Economics, Wuhan Textile University, Wuhan 430200, China; hsun@wtu.edu.cn (H.S.); liwjing2007@163.com (W.L.); 2 Center of Industrial Economy, Wuhan Textile University, Wuhan 430200, China; 3 School of Economics, Guizhou University, Guiyang 550025, China; 4 China Karst Rural Revitalization Research Institute, Guizhou University, Guiyang 550025, China; 5 School of Cyber Science and Engineering, Huazhong University of Science and Technology, Wuhan 430074, China; junfeng@hust.edu.cn

**Keywords:** social poverty line, rural relative poverty, urban relative poverty, regional comparison, income distribution

## Abstract

China achieved comprehensive poverty eradication under the current standards in 2020, but eliminating absolute poverty does not mean the end of poverty alleviation and reduction; relative poverty will exist for a long time and has become the subject of poverty study. In this paper, the social poverty line (SPL) index is utilized to establish the relative poverty standard, and CHFS2017 is used to compare the regional distribution of relative poverty in China. The results show that the relative poverty in rural areas is more serious than that in urban areas. The rural relative poverty rate in five provinces and cities including Beijing is over 60%, and the rural relative poverty rate in Qinghai is low. The urban relative poverty rate in many provinces and cities of the central and western regions is below 40%, and the relatively high relative poverty rate in the eastern region has drawn attention to the issue of the income distribution. Moreover, a logit model for binary is employed for the influencing factor analysis of the relative poverty of urban and rural residents. The results show that the education year has a negative effect on the relative poverty of urban and rural residents. Happiness has a positive effect on urban residents, government financial expenditure and financial support for agriculture have different effects on rural residents and urban residents. Therefore, we put forward aiming at relative poverty in the rural areas of the central and western regions to reduce financial pressure and increase the benefits of poverty reduction.

## 1. Introduction

Since the reform and opening up, the anti-poverty struggle in rural China has won a huge victory with economic development. In 2020, China achieved comprehensive poverty eradication under the current standards, but eliminating absolute poverty does not mean the end of poverty alleviation and reduction, and relative poverty will exist for a long time (Wan et al., 2021) [1]. In October 2019, the fourth Plenary Session of the 19th CPC Central Committee officially raised the issue of addressing relative poverty (Wang et al., 2020) [2]. Although developed countries have adopted many policy practices to alleviate relative poverty, because China is different from developed countries in its special national conditions, the measurement and strategies of relative poverty are not compatible. However, for the definition of relative poverty direction, all researchers agreed that relative poverty is defined as follows: the income can reach or exceed subsistence and basic development requirements and still be in a lower standard of living compared with the social and economic development level in a certain period (Foster, 1998) [3]. This definition, to a great extent, reflects the status of the unfair distribution of social (Hagenaaars, 2017) [4].

The setting of the poverty line is the starting point for studying poverty (Sen, 1999) [5], because how the poverty line is set is directly related to the identification of poor people, the analysis of causes of poverty, and even the formulation of poverty alleviation and reduction policies (Ravallion, 2011) [6]. Since the implementation of the seven-year Poverty Alleviation Plan, China has established a rural poverty monitoring system and has formulated a national rural poverty standard, which has been adjusted accordingly at different stages of poverty alleviation. China’s poverty-reduction work has gradually shifted from eliminating absolute poverty to alleviating relative poverty, and the poverty standard has also shifted from absolute to relative. Absolute poverty is the inability to meet a specific basic need (food and non-food) and is generally measured by income or consumption. Relative poverty means that some people are poorer than others or have incomes that are much lower than the average. With economic and social development, some countries have gradually eliminated absolute poverty and have started to employ relative standards to measure poverty. China has eliminated absolute poverty under the current standard in 2020, and the formulation of the relative poverty standard is an important issue in current and future poverty-alleviation research. In 1976, OECD proposed to take 50% or 60% of the median or average income of a country or region as the poverty line. This is also a method commonly used in relative poverty measurement at home and abroad at present (Alkire, and Santos, 2014) [7]. In 1979, British researchers began to put forward the concept of relative poverty and used the standard of “60% of median family income” to measure poverty. The World Bank considers members of society with less than one-third of the average income to be relatively poor. The EU member classifies people with less than 60% or 50% of the median income as relatively poor. In the United States, in 2017, the standard is USD 24,600 for a family of four and USD 20,420 for a family of three (Alkire, and Foster, 2011) [8]. Singapore defines relative poverty as those in the bottom 20 percent of household income, while Japan defines relative poverty as those whose income does not reach 50 percent of middle-income households (Moller et al., 2003) [9]. Some of China’s more developed provinces have already reflected this basic idea. Zhejiang province set its poverty line to CNY 4600 as early as 2012, twice the national standard at that time, and Jiangsu province also proposed to set its poverty line to CNY 6000. Some researchers have pointed out that the current poverty line in China is higher than the extreme poverty line in the world, so it is suggested to adopt a certain proportion of median income to determine the relative poverty line and gradually increase this proportion with the improvement of economic development (Ravallion, and Chen, 2019) [10]. Additionally, some researchers put forward, considering the hybrid characteristics of absolute poverty and relative poverty during the transformation, that the relative line on the basis of “Martin method” with certain floating space can be formulated; or considering regional differences between urban and rural areas, according to the median income, the urban and rural relative poverty lines first are formulated, respectively, and then convergence is gradually adjusted (Christiaensen, and Kanbur, 2017) [11]. The median method is still the mainstream method of relative poverty measurement, but this method has some defects: it cannot measure the degree of social relative poverty, but only measures the incidence of social relative poverty. A composite index to measure relative poverty was adopted (Takayama, 1979) [12]. First, the ratio between the weighted income level of the top 40% of earners and the weighted average income level of the earners below 60% was used to measure the relative poverty level of the society. At the same time, the ratio of the population at the average level of 60% to the total population is used to measure the incidence of social relative poverty (Wang et al., 2021) [13]. Finally, the product of the two is used as the index to measure relative poverty. There is no doubt that people’s perception of relative poverty also changes with the development of the social economy, but income or consumption as a way to measure is commonly used. Some researchers also measure relative poverty by using income, education, health, employment, social security, living environment, and multidimensional measurements (Alkire, 2007; Wagle, 2005) [14,15]. Researchers have proposed poverty-measurement methods from different dimensions, but it is rare to measure the overall relative poverty in China in practice.

In order to better manage relative poverty, we must first identify the relative poor population. In this paper, the social poverty line (SPL) adopted by the World Bank is used to measure the relative poverty of urban and rural residents in China. Firstly, the SPL index proposed by Jolliffe and Prydz et al. is used to formulate the relative poverty line of urban and rural areas in each region. Then, the regional distribution of relative poverty in China is analyzed by using the survey data of large samples in China. Finally, the influencing factors of relative poverty are analyzed. Compared with the existing literature, the main contributions of this paper are as follows: first, due to China’s vast territory, the regional economic development varies greatly, and the duality of urban and rural development is significant [16]. Therefore, the social poverty index is selected to formulate the relative poverty standards of urban and rural areas of provinces and cities, respectively, which is different from the traditional “one line across the country” based on national income (Sun et al., 2020; Yang et al., 2015) [17,18]. This can more accurately identify China’s relative poverty population and measure China’s relative poverty situation. Secondly, we show the comparative analysis of the relative poverty distribution in China. The research on absolute poverty is abundant, but the research for the measurement of relative poverty in a large area of the country is relatively limited. The measurement and regional distribution analysis of relative poverty in the whole country can provide a good foundation and guarantee for the subsequent treatment of relative poverty. Thirdly, analyzing the influencing factors of relative poverty can provide valuable suggestions to relevant departments of relative poverty management.

The remaining parts of this paper are arranged as follows: we review the changes in China’s poverty standard and put forward the social poverty standard SPL in Section 2. We measure the relative poverty of urban and rural residents in China and make a regional comparative study in Section 3. Section 4 shows the analysis of influencing factors of relative poverty. We discuss the influencing factors of relative poverty in China as a whole, as well as urban and rural relative poverty, and other relative poverty standards with the national per capita consumption measurement are used to test the robustness of the above results. Finally, we put forward research conclusions and policy recommendations in Section 5.

## 2. Change of Poverty Standard and Social Poverty Standard SPL in China

In 1986, in accordance with the World Bank Law on Basic Needs, the state set China’s first rural poverty standard based on the income and expenditure survey data of 67,000 rural households across the country, which includes two parts: food consumption expenditure to maintain basic life and non-food consumption expenditure (clothing, housing, transportation, fuel, supplies, medical treatment, education, and entertainment, etc.) (Ravallion, 2010) [19]. Food expenditure is calculated and added up from a basket of basic food consumption and corresponding prices using the minimum nutritional requirement of 2100 calories per person per day. Non-food consumption expenditures are measured in two ways: the first is the low poverty line measured by the Martin method, which represents minimum non-food needs, such as essential spending on basic clothing and heating. The low poverty line can only support basic food and clothing, and food consumption accounts for as much as 70 percent. The second is the high poverty line based on the Engel coefficient method, including necessary food, clothing and housing, as well as necessary education, health and other expenditures. On the basis of determining the Engel coefficient of poverty-stricken peasant households, the non-food consumption expenditure and the total consumption expenditure are deducted according to the food consumption expenditure. The high poverty line attaches more importance to non-food consumption than the low poverty line, and the proportion of non-food consumption expenditure is 40%, representing a stable level of food and clothing (Zheng, 2001) [20]. Before 2008, China determined the poverty line, respectively, on the basis of the Martin method and Engel coefficient method, calling it the “poverty line” and “low-income line”. In 2007, the rural poverty line was CNY 785 and the low-income line was CNY 1067. By the end of 2007, 14.79 million people lived in absolute poverty in rural areas, with a poverty rate of 1.6 percent. The low-income population is 284.11 million, accounting for 3.0% of the rural population (Rural Social and Economic Survey Department, China’s National Bureau of Statistics, 2008). After 2008, the high poverty line based on the Engel coefficient method was adopted as the rural poverty standard. According to this method, China’s rural poverty standard in 1985 was CNY 205 per capita annual net income, which has been adjusted year by year since then according to the changes in the price index. A rural poverty monitoring system was set up in 1997 to monitor poverty-stricken areas. In 1990, 1994, 1997, and 2010, the Rural Socio-Economic Survey Team of China’s National Bureau of Statistics determined the rural poverty standard based on household data from the National Rural Household Survey and updated it based on the rural consumer price index in other years. In 2009, China raised the rural poverty standard to an annual per capita net income of CNY 1196. In 2011, the Central Conference on Poverty Alleviation issued the Outline for China’s Rural Poverty Alleviation and Development (2011–2020), which defined the task of poverty alleviation and development as “consolidating the achievements of subsistence and clothing, accelerating poverty alleviation and prosperity, improving the ecological environment, enhancing development capacity, and narrowing the development gap”. In order to narrow the development gap and allow more poor people to share in the fruits of reform and opening up, the central government has set the poverty alleviation standard to CNY 2300 per capita net income of rural residents per year (in 2010, the price did not change; this approximately USD 361), and the number of people living in poverty in rural areas rose from 26.88 million to 128 million. According to China’s current rural poverty standard (CNY 2300 per person per year at 2010 prices), 770 million people lived in poverty in rural China in 1978, and the poverty rate was 97.5 percent. By the end of 2018, 16.6 million people lived in poverty in rural areas, which is 750 million fewer than that in 1978. The poverty rate is 1.7%, which is 95.8 percent lower than that of 1978. With the development of the economy and society, the poverty standard has gradually changed, and the relative poverty standard has become the mainstream of poverty measurement after 2020.

Most low- and middle-income countries define poverty as the inability to meet basic needs. Absolute poverty lines reflect a fixed level of material welfare, while relative poverty is a concept that increases with a country’s economic development. Townsend (1979) [21] believed that basic needs are not always fixed. Sen (1983) [22] fixed poverty at the lowest functional level of a society, that is, the poverty standard was determined according to the expenditure required by the lowest functional level of the society. However, countries at different levels of economic development spend different amounts of money to meet basic needs. For example, in some backward areas, basic food and clothing can satisfy the minimum social function, while in some developed areas, participation in the labor market may require Internet, mobile phones, computers, etc. Atkinson and the World Bank agreed that global poverty should be measured by the proportion of the total population of a society (World Bank, 2017, 2018) [23,24]. Therefore, based on the works of Atkinson and Bourguignon (2001) [25] and Ravallion and Chen (2010, 2013) [26,27], Jolliffe and Prydz (2017) [28] proposed the Social Poverty Line (SPL) for measurement, which can better assess the relative poverty situation of regions with different levels of economic development. Ravallion and Chen (2011) adopted the Max (Z*, α + *kM*) poverty measurement method, where Z* is the floor, the minimum poverty level, and the absolute poverty line; α is a fixed intercept, and kM explains the relativist part of the upward tilt in the level of economic development. SPL = Max (Z*, α + *kM*); the implication is that the poverty line is determined by the greater value between the absolute poverty line Z* and the relative poverty line α + *kM*, but the relative poverty line is not extremely low because the average income of the country is very low but has a minimum threshold α. Atkinson-Bourguignon and Ravallion-Chen suggested that Z* is an international poverty line of USD 1 per day (1985 PPP) or USD 1.25 per day (2005 PPP), α = 0.6 and *k* = 0.33; M is per capita consumption expenditure in the national economy. Based on the existing works such as Sun et al., (2018) [29], Decerf (2021) [30], Chen and Ravallion (2021) [31], and Garroway and de Laiglesia (2012) [32], this paper applies the relative poverty line standard proposed by Jolliffe and Prydz (2017) [33]:

SPL = Max (USD 1.9, USD 1.0 + 0.5 × Median Consumption).(1)

China is a vast country with great regional differences. It is difficult to measure relative poverty in China comprehensively by using a ruler across the country. The long-term existence of the urban-rural dual economic structure has also brought two serious consequences to the adoption of the unified relative poverty line: ignoring the relative poverty in urban areas and the large rural relative poverty population has brought great financial pressure (Zhang et al., 2022) [34]. This paper plans to delimit relative poverty standards for each province and city, respectively, and adopts different standards for rural and urban areas to measure and compare regional relative poverty.

## 3. Measurement and Regional Comparison of Relative Poverty in China: Based on the SPL Method

The SPL index used in this paper starts with an intercept of USD 1 per day (purchasing power level in 2011, the same below) and a gradient of 50% of median consumption or income, at the same time, considering the international poverty standard of USD 1.90 a day, USD 1.90 is taken as a fixed element, and 50% of the mean consumption (or income) is taken as the relative poverty element. This proportion method can not only meet the consistent relative poverty standards of countries at different levels but is also consistent with the United Nations sustainable development goals SDG10.2.1. According to Equation (1), the relative poverty line of Chinese residents in 2019 can be calculated as CNY 13,634.94. The urban relative poverty line is CNY 16,887.19, and the rural relative poverty line is CNY 9519.35. There is a large gap between urban and rural relative poverty lines. The urban relative poverty line is about 1.77 times higher than the rural poverty line, which is much higher than China’s current absolute poverty line.

This paper is based on the data from the National Bureau of Statistics of China and the China Household Finance Survey in 2017 (CHFS2017). CHFS2017 is the fourth round of the survey conducted by the Survey and Research Center for China Household Finance, covering 29 provinces (autonomous regions and municipalities), 355 districts and counties, and 1428 village (neighborhood) committees. The number of sample households is 40,011, and the data is representative of national, provincial and sub-provincial cities. The demographic characteristics of the CHFS data are highly consistent with those published by China’s National Bureau of Statistics; in terms of the family size, age structure and sex ratio of the population, it is relatively consistent with the data of the National Statistical Office. In terms of total resident income, CHFS is quite consistent with the total resident income, total urban and rural resident income and per capita income released by China’s National Bureau of Statistics. It shows that the CHFS sample is fully representative of the whole country.

In the China Household Finance Survey data (CHFS2017), the data focused on consumption or income as the statistics of 2016. CHFS2017 can be used to calculate the relative poverty lines of rural and urban areas in various provinces and cities, and then calculate the relative poverty population. In 2016, the relative poverty standard of China’s overall residents is CNY 11,237.99, that of urban residents is CNY 14,221.94, and that of rural residents is CNY 7747.38, far higher than the absolute poverty standard of CNY 2300; they are 65.68%, 61.62% and 76.48% of the corresponding per capita consumption, respectively. This is not far from the international standard of the relative poverty of 60 percent of median income or consumption. According to the data of the China Household Finance Survey, the per capita consumption of Chinese residents is CNY 21,528, the per capita consumption of Chinese rural residents is CNY 11,476, and the per capita consumption expenditure of urban residents is CNY 26,220. According to China’s National Bureau of Statistics, per capita consumption in China is CNY 17,111, CNY 23,079 in urban areas and CNY 10,130 in rural areas. CHFS2017 is uniformly higher than the consumption level calculated by China’s National Bureau of Statistics (see Table 1).

Table 2 shows the per capita consumption expenditure in urban and rural areas of various provinces and cities in China calculated according to the data of China’s National Bureau of Statistics. In 17 provinces and cities, rural per capita consumption is higher than the national average. There are nine provinces and cities in the eastern region: Beijing, Tianjin, Shanghai, Jiangsu, Zhejiang, Fujian, Shandong, Guangdong, and Hainan. There are three provinces and cities in the central region: Jiangxi, Hubei, and Hunan. There are three provinces and cities in western China: Inner Mongolia, Sichuan, and Qinghai. There are two in northeast China: Liaoning and Heilongjiang. In 14 provinces and cities, rural per capita consumption is lower than the national level. There is Hebei in the eastern region and Shanxi, Anhui, and Henan in the central region. There are nine provinces and cities in the western region: Guangxi, Chongqing, Guizhou, Yunnan, Tibet, Shanxi, Gansu, Ningxia, and Xinjiang. There is Jilin in northeast China. The per capita urban consumption in 17 provinces and cities is higher than the national level: Beijing, Tianjin, Shanghai, Jiangsu, Zhejiang, Fujian, Shandong, and Guangdong of the eastern region; Henan, Hubei, and Hunan of the central region; Inner Mongolia, Chongqing, Shaanxi, Ningxia of the western region; and Liaoning in the northeast region. The per capita urban consumption level in 14 provinces and cities is lower than the national per capita urban consumption level: Hebei in the eastern region; Shanxi, Anhui, Jiangxi in the central region; Guangxi, Sichuan, Guizhou, Yunnan, Tibet, Gansu, Qinghai, and Xinjiang in the western region and Jilin; and Heilongjiang in the northeast region. Most residents’ per capita consumption level is higher than the national average in the eastern provinces and cities, both urban and rural, while there are many residents from western provinces and cities whose per capita consumption level is lower than the national average in both rural and urban, which is consistent with the national regional economic development.

Table 3 shows the relative poverty standards of provinces and cities in China. This standard is calculated based on the per capita consumption level of all provinces and cities in China, so its distribution is the same as the analysis in Table 2. The average relative poverty standard of China’s cities falls in between 40% of the median per capita disposable income and 50% of that calculated by some scholars (Wang et al., 2021) [35]. The average relative poverty standard in rural areas is about 60% of the median per capita disposable income.

Under the SPL standard based on the per-capita consumption of each province and city, the total number of relatively poor people in China is 19,119, 3620 more than 15,499 under the SPL standard based on national per capita consumption. This again demonstrates the high accuracy of subregional calculations of relative poverty. Relative poverty identification I’s relative poverty identification is more accurate; relative poverty identification I is to calculate the relative poverty standards based on the average per capita consumption level of each province and then identify the relative poverty population, and it can identify more relatively poor people. Similarly, the situations of urban and rural areas are the same. The number of urban relative poverty population under relative poverty identification I is 12,630. This is 3021 more than the number of relative poverty population under relative poverty identification II, which is 9609. Relative poverty identification II is to calculate the relative poverty standard based on the national average per capita consumption level and then identify the relative poverty population. The number of rural relative poverty population under relative poverty identification I and II is 6489 and 5890, respectively. According to the relative poverty identification criteria I and II, there are 14,863 relatively poor people, and the number of urban and rural relatively poor people is 9289 and 5565, respectively (see Table 4).

**Figure 1:**
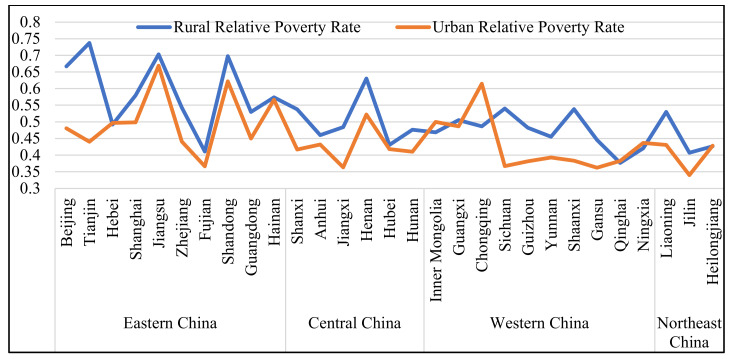
Incidence of relative poverty in urban and rural areas across the country.

**Figure 2:**
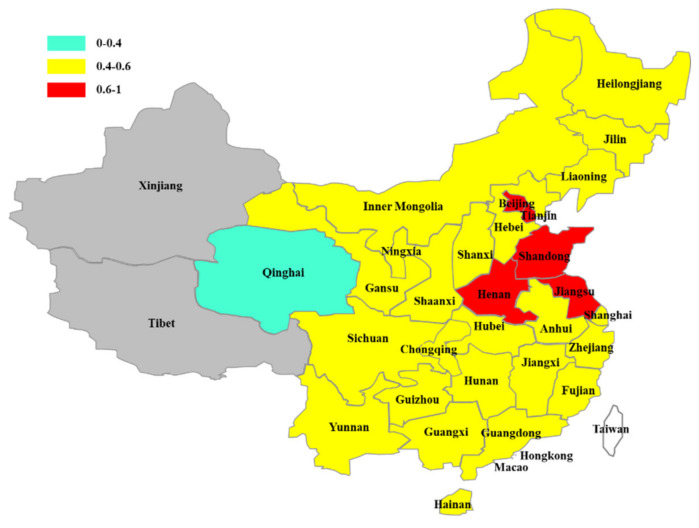
Rural relative poverty rate of provinces and cities in China.

**Figure 3:**
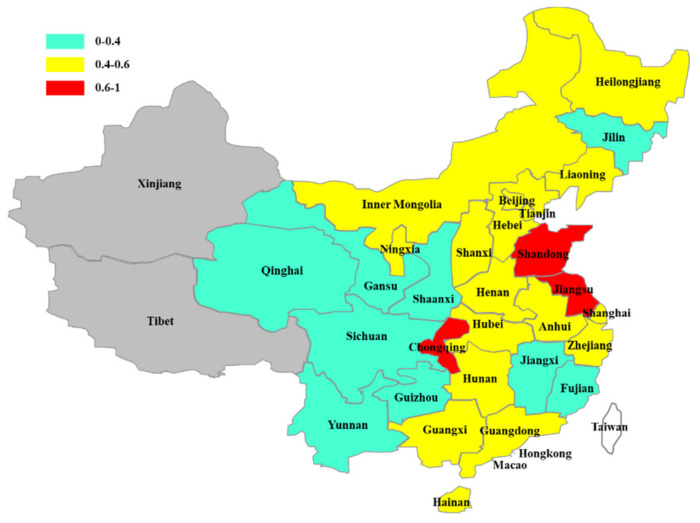
Urban relative poverty rate of provinces and cities in China.

By comparing the relative poverty rate between urban and rural areas, it can be found that the rural relative poverty rate is higher than the urban relative poverty rate in most provinces and cities of China (See Figure 1). Except Chongqing, Hebei, Inner Mongolia, Qinghai, Ningxia and Heilongjiang (the rural relative poverty rate is lower than the urban relative poverty rate), the rural relative poverty rate of the rest is higher than the urban poverty rate (See Table 5). This is consistent with what we have observed. The relative poverty rate is ranked in a different order from absolute poverty. The order from high to low is as follows: eastern region, central region, and western region. It reflects that the income gap between the rich and the poor is larger in the developed area, leading to a higher relative poverty rate in this region. The average rural relative poverty rate in eastern China is 0.593, while the urban relative poverty rate was 0.503. The rural and urban relative poverty rates are 0.503 and 0.427 in central China, and 0.472 and 0.431 in western China, respectively. Seven provinces and cities, including Beijing, Tianjin, Shandong, Henan and Jiangsu, have a rural relative poverty rate of more than 6 percent, and four of them are in eastern China. Qinghai was the only province where the rural relative poverty rate is 4%, while the rural relative poverty rate in other provinces is between 0.4% and 0.6% (See Figure 2). Relative poverty in urban areas is less severe than in rural areas. In Figure 3, there are many provinces and cities where the urban relative poverty rate is below 4%, namely Qinghai, Gansu, Shaanxi, Sichuan, Guizhou, Yunnan, Jiangxi, Fujian, and Jilin. There are three provinces and cities with an urban relative poverty rate of more than 6%, two of which are in the eastern region, namely Shandong, and Jiangsu. The rest of the provinces and cities have an urban relative poverty rate of between 4% and 6%. From the distribution of relative poverty incidence, although the absolute poverty level in eastern China is low, the relative poverty rate is high. The eastern region has a high per capita income and consumption level, and its relative poverty standard is relatively high, so there are a large number of relatively poor people. This also reflects that the income distribution structure of the population in the eastern region is a problem that needs to be focused on in the process of relative poverty governance.

## 4. Analysis of the Influencing Factors of the Relative Poverty of Urban and Rural Residents in China

Through the above analysis, we can clearly see the distribution of relative poverty in urban and rural areas of China. But why are there regional differences in relative poverty? What is the cause of this distribution? This section analyzes the influencing factors of the relative poverty of urban and rural residents in China and answers the above questions. To do this, we consider a logit model for binary responses:

P[*y* = 1|*X*] = G(*β*_0_ + *β*_1_ × 1 + … + β*_k_* × *k*) = G(*β*_0_ + *Xβ*) (2)

where *y* is the variable capturing whether the resident is in relative poverty (value is 0 or 1), and *X* represents the set of all explanatory variables. For any real number *z*, G is a function strictly between 0 and 1: G(*z*) = $\frac{\exp\left(z\right)}{1+\;\exp\left(z\right)}$.

The explanatory variables are the variable capturing whether the resident lives in relative poverty, the variable capturing whether the rural resident lives in relative poverty, and the variable capturing whether the urban resident lives in relative poverty. Firstly, it is necessary to control individual characteristic variables, such as age, gender, years of education, marital status, health status, and happiness, which can all have a certain impact on poverty. To confirm that the answers of the respondents were acceptable and robust, only samples for residents aged 16 to 100 were included (Chen et al., 2020 [36], Zhang et al., 2020 [37]), and the observations with missing information were excluded. Therefore, the size of the final samples used in the data analysis of this paper was 39,036. China is a society based on family. In addition to individual characteristics, family factors also affect poverty (Benjamin et al., 2006) [38], for example, per capita household income, proportion of agricultural income, household consumption, etc., and external environment including the family environment of an individual can also have an impact on poverty, such as regional financial support policies (Wang et al., 2020) [39]. Based on the above considerations, the following variables are selected in this paper (see Table 6).

As can be seen from the first column of regression analysis results of the impact of various variables on relative poverty in Table 7, the effect of age on relative poverty of residents presents an inverted U-shaped relationship. That is, when young, the relative poverty increases with the increase of age (no wealth accumulation, large family population, heavy burden), but after reaching a certain age, the relative poverty decreases with the increase of age. With the age, wealth accumulation increases and the probability of relative poverty decreases. The relative poverty degree of males is higher than that of females, and the longer the years of education are, the lower the probability of relative poverty is, which fully reflects the role of human capital. Married people have a higher relative poverty rate than unmarried people, which may result from larger family size, heavier burden, and lower per capita income (consumption). The higher the per capita household income is, the lower the relative poverty rate is. The higher the proportion of agricultural income in total household income is, the lower the relative poverty rate is, mainly for the rural poor. Agricultural income is their main source of income, therefore the higher the income is, the lower the relative poverty rate is. The higher the proportion of tobacco and alcohol expenditure in total household expenditure is, the higher the relative poverty rate is. The more you spend on alcohol and tobacco, the more you spend on living, and the more likely you are to fall into poverty. Expenditure on tobacco and alcohol may be used as consumption expenditure in daily life or expenditure on gift exchange, but they all lead to the same poverty result. Some researchers classify the population with such poverty phenomenon as expenditures-type poverty (Hulme, and Shepherd, 2003) [40]. The higher the local fiscal expenditure is, the higher the relative poverty rate is, and the explanation is the same as that of the higher relative poverty rate of urban residents in eastern China before. The higher the proportion of fiscal support for agriculture is, the lower the relative poverty rate is. Fiscal support for agriculture benefits the relative poverty population and reduces the relative poverty rate. It can be seen from the regression results of the relative poverty of rural residents in the second column of Table 7 that the influence of age on relative poverty is U-shaped, but not significant. In rural areas, there is no significant correlation between relative poverty and age, and the relatively poor people are scattered in all age groups. The relative poverty rate of males is higher, and the higher the number of years of education is, the lower the relative poverty rate is, which reflects the positive effect of education. Married people have higher relative poverty rates than unmarried people. The higher the rural health status is, the higher the relative poverty rate is, but not significantly. The influence of variables is basically the same as that of the national population. The influencing factors of urban relative poverty can be seen in the third column of Table 7. The influence of age on the relative poverty of urban residents shows an inverted U-shaped relationship. The better the health status gets, the lower the relative poverty rate of urban residents is, but not significantly. The happier people are in towns, the higher their relative poverty rate is. This conclusion is consistent with other researchers’ research, which does not necessarily mean that more money leads to happiness (Boyce et al., 2010) [41]. Other factors’ effects are the same as the whole country. The last column is the relative poverty population identified based on the SPL standard under the national per capita consumption level. This poverty identification may be less stringent than the explained variables in the first column, but it is still a method used as a robustness test. The regression result is basically the same as the previous one. All variables have the same influence direction, but the regression coefficient is slightly different, which proves that the regression result mentioned above is robust.

Through the relative poverty of urban and rural factors regression results, we found that other variables had little difference in influence, except health and happiness (Amato, and Zuo, 1992; Dowling, and Yap, 2012) [42,43]. In urban areas, the higher the level of health, the lower the likelihood of relative poverty, that is, medical treatment in urban areas has a greater impact on residents’ lives and is the main factor leading to poverty. In rural areas, the higher the health level is, the higher the likelihood of relative poverty is, probably because of the lower income in rural areas, the proportion of people who spend more money on medical treatment is much lower than in urban areas. The happier urban residents are, the higher the relative poverty rate is, while the happier rural residents are, the lower the relative poverty rate is. This may be because rural and urban residents are at different levels of Maslow’s hierarchy of needs.

## 5. Conclusions

In this paper, the social poverty line (SPL) is selected to establish the relative poverty standard of all provinces and cities in China, and the large sample data of the China Household Finance Survey is used to identify the urban and rural relative poverty population of all provinces and cities in China firstly. Then the influencing factors of urban and rural relative poverty in each province are analyzed. Finally, the relative poverty population is identified by the SPL standard under the national per capita consumption level, and the robustness test is carried out. The results show that relative poverty in rural areas is more serious than in urban areas. The rural relative poverty rate in Beijing, Tianjin, Shandong, Jiangsu and Henan provinces is above 60%. The rural relative poverty rate in other provinces and cities is between 40% and 60%, except Qinghai province, where the rural relative poverty rate is below 40%. Qinghai, Gansu, Shaanxi, Sichuan, Yunnan, Guizhou, Jiangxi, Fujian and Jilin have more urban relative poverty rates below 40 percent. Chongqing, Shandong and Jiangsu have urban relative poverty rates above 60 percent, while the rest of the provinces and cities have urban relative poverty rates between 40 and 60 percent. The urban-rural dual structure can explain the difference between urban and rural relative poverty, and the income distribution gap can explain the high relative poverty degree of some provinces and cities (Fan et al., 2002) [44]. Improving the level of education is an important means to eliminate poverty. Medical security for residents is the foundation of social development. Promoting national fitness and ensuring good health is the guarantee of economic development. It is better to keep a good attitude and increase happiness. We will increase fiscal support for agriculture to provide the relative poverty population benefit. Through social care, increasing publicity and other means, we will promote the increase in income of low-income groups and improve the urban-rural dual structure and uneven income distribution within cities and villages.

The corresponding policy implications are as follows. Relative poverty is also a matter of concern. However, the way to reduce relative poverty is different from the way to eliminate absolute poverty. Relative poverty is no longer simply income poverty but entails multidimensional poverty including food, clothing, housing, education, recreation, health, society communication and capability. Therefore, we should pay attention to multiplex development, not only income, but also employment opportunities, personal ability and quality, development opportunities and so on. The surrounding environment and hardware are the foundation for personal development. The government needs to address this. Education is an important and less risky way to reduce poverty and achieve development; therefore, it is important to encourage the relatively poor through education. Health, employment, and so on should be paid attention to because relative poverty is multicomponent. The government should give more support to the young because young people have less wealth accumulation and require more spending.

The rural areas in China are not only the gathering place of absolute poverty but also the problem of relative poverty. The relatively serious rural poverty areas are mainly economically developed areas and large agricultural provinces, which provide more sources of income increase. Most of the provinces that have just emerged from absolute poverty are ones with low rural relative poverty rates. It means that with the development of the economy and the comprehensive elimination of absolute poverty, the problem of relative poverty is becoming more and more prominent. Because the properties and causes are different between absolute poverty and relative poverty, the approach to eliminating relative poverty is also different from absolute poverty. It is the prerequisite for reducing relative poverty to know the extent of relative poverty. Moreover, we should know more influencing factors of relative poverty. In our paper, we just discussed the influencing factors on the whole, but the exact mechanism is not well understood; therefore, one of our future works is to study this specific problem. With economic development, the degree and characteristics of relative poverty will change; hence, we should put forward updating measures of relative poverty.

## Figures and Tables

**Table 1:** Per capita consumption data from China’s National Bureau of Statistics and CHFS.

	Per Capita Consumption Level of Chinese People	Per Capita Consumption of Urban Residents	Per Capita Consumption of Rural Residents
CHFS	21,528	26,220	11,476
State Statistics Bureau	17,111	23,079	10,130

**Table 2:** Per capita consumption expenditure of urban and rural residents in provinces and cities of China in 2016 (CNY).

Province and City	Rural Per Capita Consumption	Urban Per Capita Consumption	Province and City	Rural Per Capita Consumption	Urban Per Capita Consumption
National	10,130	23,079	Henan	9291	23,454
Beijing	24,285	52,721	Hubei	10,860	25,703
Tianjin	22,194	39,181	Hunan	10,461	24,025
Hebei	8897	19,276	Guangdong	14,784	34,667
Shanxi	9226	19,724	Guangxi	8225	22,491
Inner Mongolia	13,013	28,289	Hainan	10,512	24,664
Liaoning	12,145	29,254	Chongqing	9433	28,209
Jilin	8390	18,144	Sichuan	11,094	21,246
Heilongjiang	10,305	22,318	Guizhou	8887	22,301
Shanghai	23,660	53,240	Yunnan	8336	22,365
Jiangsu	23,459	41,957	Tibet	5952	18,775
Zhejiang	22,028	35,152	Shaanxi	8768	23,206
Anhui	8565	22,030	Gansu	6781	21,128
Fujian	15,653	27,859	Qinghai	10,505	22,761
Jiangxi	11,320	20,335	Ningxia	9980	25,384
Shandong	15,970	33,016	Xinjiang	8816	22,272

**Table 3:** Relative poverty lines of provinces and cities in China SPL (CNY).

Province and City	SPL in Rural Areas	SPL in Cities	Province and City	SPL in Rural Areas	SPL in Cities
National	7747.381	14,221.94	Henan	7327.99	14,409.49
Beijing	14,824.99	29,042.99	Hubei	8112.49	15,533.99
Tianjin	13,779.49	22,272.99	Hunan	7912.99	14,694.99
Hebei	7130.991	12,320.49	Guangdong	10,074.49	20,015.99
Shanxi	7295.491	12,544.49	Guangxi	6794.99	13,927.99
Inner Mongolia	9188.991	16,826.99	Hainan	7938.49	15,014.49
Liaoning	8754.991	17,309.49	Chongqing	7398.99	16,786.99
Jilin	6877.491	11,754.49	Sichuan	8229.49	13,305.49
Heilongjiang	7834.991	13,841.49	Guizhou	7125.99	13,832.99
Shanghai	14,512.49	29,302.49	Yunnan	6850.49	13,864.99
Jiangsu	14,411.99	23,660.99	Tibet	5658.49	12,069.99
Zhejiang	13,696.49	20,258.49	Shaanxi	7066.49	14,285.49
Anhui	6964.991	13,697.49	Gansu	6072.99	13,246.49
Fujian	10,508.99	16,611.99	Qinghai	7934.99	14,062.99
Jiangxi	8342.491	12,849.99	Ningxia	7672.49	15,374.49
Shandong	10,667.49	19,190.49	Xinjiang	7090.49	13,818.49

**Table 4:** Number of people living in relative poverty in urban and rural areas (person).

	Number of People Living in Relative Poverty	Relative Poverty in Urban Areas	Relative Poverty in Rural Areas
Relative poverty identification I	19,119	12,630	6489
Relative Poverty identification II	15,499	9609	5890
Relative poverty identification I and II	14,863	9289	5565

**Table 5:** Number and ratio of the rural and urban relative poverty population in provinces and cities of China (person, %).

Province and City	Rural Areas	Cities	Province and City	Rural Areas	Cities
National	6489/12,732 ^1^ (0.510 ^2^)	12,630/27,279 (0.463)	Shandong	441/632 (0.698)	942/1515 (0.622)
Beijing	68/102 (0.667)	621/1293 (0.480)	Henan	331/525 (0.630)	324/621 (0.522)
Tianjin	45/61 (0.738)	443/1006 (0.440)	Hubei	242/562 (0.431)	434/1038 (0.418)
Hebei	297/604 (0.492)	485/976 (0.497)	Hunan	272/571 (0.476)	416/1014 (0.410)
Shanxi	384/714 (0.538)	316/758 (0.417)	Guangdong	372/702 (0.530)	1010/2245 (0.450)
Inner Mongolia	105/224 (0.469)	137/274 (0.500)	Guangxi	146/289 (0.505)	269/553 (0.486)
Liaoning	257/485 (0.530)	754/1750 (0.431)	Hainan	179/312 (0.574)	299/528 (0.566)
Jilin	249/612 (0.407)	287/844 (0.340)	Chongqing	202/415 (0.487)	598/973 (0.615)
Heilongjiang	154/361 (0.427)	415/969 (0.428)	Sichuan	349/646 (0.540)	398/1085 (0.367)
Shanghai	40/69 (0.580)	929/1864 (0.498)	Guizhou	190/394 (0.482)	125/328 (0.381)
Jiangsu	301/428 (0.703)	931/1392 (0.669)	Yunnan	244/536 (0.455)	188/479 (0.392)
Zhejiang	371/683 (0.543)	727/1650 (0.441)	Shaanxi	176/327 (0.538)	354/924 (0.383)
Anhui	249/541 (0.460)	202/468 (0.432)	Gansu	148/332 (0.446)	178/492 (0.362)
Fujian	306/745 (0.411)	378/1032 (0.366)	Qinghai	110/292 (0.377)	168/439 (0.383)
Jiangxi	169/349 (0.484)	167/460 (0.363)	Ningxia	92/219 (0.420)	135/309 (0.437)

^1^ A denotes the total number of people living in relative poverty in the region, and B refers to the total population of the region in A/B. ^2^ The numbers in brackets represent the incidence of relative poverty, (total relative poverty in the region)/(total population in the region) × 100%.

**Table 6:** Statistical analysis of each variable.

Variables	Variable Definition	Mean	Standard Deviation	Minimum	Maximum
Relative poverty identification I ^1^	1 = yes, 0 = no	0.478	0.500	0	1
Relative Poverty identification II ^2^	1 = yes, 0 = no	0.387	0.487	0	1
Rural relative poverty identification	1 = yes, 0 = no	0.510	0.500	0	1
Urban relative poverty identification	1 = yes, 0 = no	0.463	0.499	0	1
Age	Age of resident (2017)	55.202	14.213	16	100
Age squared × 10^−3^		3.250	1.583	0.256	10
Gender	1 = male, 0 = female	0.793	0.405	0	1
Education years	0 = did not go to school, 6 = primary school, 9 = junior high school, 12 = senior high school, 12 = technical secondary school/vocational high school, 15 = junior college/vocational high school, 16 = undergraduate course, 19 = master graduate student, 23 = doctor graduate student	9.276	4.168	0	23
Marital Status	1 = yes, 0 = no	0.848	0.359	0	1
Physical condition	1 = very bad, 2 = bad, 3 = fair, 4 = good, 5 = very good	3.387	1.016	1	5
Happiness	1 = very unhappy, 2 = unhappy, 3 = so-so, 4 = happy, 5 = very happy	3.857	0.831	1	5
The logarithm of household per capita income	The logarithm of household per capita income	10.621	1.526	−2.288	15.425
Agricultural income share	The share of agricultural income in total household income	0.125	1.009	−60.5102	127.662
Percentage of average monthly expenditure on alcohol and tobacco	Monthly expenditure on tobacco and alcohol accounted for the proportion of total family expenditure	0.005	0.007	0	0.063
Regional financial expenditure supporting agriculture	The proportion of the expenditure of local finance in agriculture, forestry, and water affairs	10.947	3.432	4.353	18.966
The logarithm of regional fiscal expenditures	The logarithm of regional fiscal expenditures	8.609	0.494	7.135	9.506

^1^ Relative poverty identification I is the SPL relative poverty standard measured by the average per capita consumption level of provinces and cities of the National Bureau of Statistics, and then identifies relative poverty by comparing the per capita household consumption of residents with it. ^2^ Relative poverty identification II is based on the SPL relative poverty standard calculated by the national average per capita consumption level, and then compares the per capita household consumption of residents with it to identify relative poverty.

**Table 7:** Analysis of the influencing factors of the relative poverty of residents.

Variables	National Relative Poverty I (1)	Rural Relative Poverty (2)	Urban Relative Poverty (3)	National Relative Poverty II (4)
Age	0.024 *** (0.000)	−0.003 (0.657)	0.033 *** (0.000)	0.023 *** (0.000)
Age square	−0.169 *** (0.000)	0.110 (0.073)	−0.273 *** (0.000)	−0.184 *** (0.000)
Gender	0.074 *** (0.000)	0.108 *** (0.008)	0.127 *** (0.000)	0.102 *** (0.000)
Education years	−0.049 *** (0.000)	−0.030 *** (0.000)	−0.070 *** (0.000)	−0.051 *** (0.000)
Marital status	0.230 *** (0.000)	0.157 *** (0.000)	0.259 *** (0.000)	0.215 *** (0.000)
Physical condition	0.013 * (0.078)	0.018 (0.124)	−0.009 (0.331)	0.014 * (0.055)
Happiness	−0.002 (0.777)	−0.009 (0.486)	0.005 (0.646)	0.006 (0.484)
Household incomes per capita	−0.217 *** (0.000)	−0.199 *** (0.000)	−0.264 *** (0.000)	−0.247 *** (0.000)
Agricultural income percentage	−0.045 *** (0.000)	−0.073 *** (0.000)	−0.093 *** (0.000)	−0.064 *** (0.000)
The proportion spent on alcohol and tobacco	4.289 *** (0.000)	5.605 *** (0.000)	5.816 *** (0.000)	2.802 *** (0.004)
Government expenditure	0.118 *** (0.000)	0.152 *** (0.000)	0.106 *** (0.000)	0.103 *** (0.000)
The proportion of government funds supporting agriculture	−0.040 *** (0.000)	−0.025 *** (0.000)	−0.042 *** (0.000)	−0.044 *** (0.000)
constant	0.862 *** (0.000)	0.549 (0.122)	1.588 *** (0.000)	0.282 (0.140)
observation	39,036	12,311	26,725	39,036

Notes: ***, * show significance at 1%, and 10% probability levels, respectively; standard errors are in parenthesis.

## Data Availability

Not applicable.
